# Design and Evaluation of a Balanced Compliant Laparoscopic Grasper

**DOI:** 10.1109/JTEHM.2023.3291925

**Published:** 2023-07-03

**Authors:** Jan-Willem Klok, Roelf Postema, Asþor T. Steinþorsson, Jenny Dankelman, Tim Horeman

**Affiliations:** Department of Biomechanical EngineeringDelft University of Technology 2860 2628 CD Delft The Netherlands; Spijkenisse Medisch Centrum 72504 3201 GZ Spijkenisse The Netherlands; Reon 105 Reykjavik Iceland

**Keywords:** Laparoscopic surgery, compliant mechanisms, grasping, haptic feedback, static balancing

## Abstract

In laparoscopic surgery, quality of haptic feedback is reduced compared to conventional surgery, leading to unintentional tissue damage during grasping. From the perspective of haptics, poor mechanical design of laparoscopic instrument joints induces friction and a nonlinear actuation-tip force relation. In this study, a novel laparoscopic grasper using compliant joints and a magnetic balancer is presented, and the reduction in hysteresis and friction is evaluated. The hysteresis loop of the novel compliant grasper and two conventional laparoscopic graspers (high quality leading commercial brand and low quality unbranded grasper) were measured. In order to assess quality of haptic feedback, the lowest grasper tip load perceivable by instrument users was measured with the novel and the conventional laparoscopic graspers. The hysteresis loop measurement yielded a mechanical efficiency of 43% for the novel grasper, compared to- 25% and 23% for the Aesculap and the unbranded grasper, respectively. The forces perceivable by the user through the novel grasper were significantly lower (mean 1.37N, SD 0.44N) than those of conventional graspers (mean 2.15N, SD 0.71N and mean 2.65N, SD 1.20N, respectively). The balanced compliant grasper technology has the ability to improve the quality of haptic feedback compared to conventional laparoscopic graspers. Research is needed to relate these results to soft and delicate tissue grasping in a clinical setting, for which this instrument is intended.

**Nomenclature**

$E_{\text {open }}$
: Energy needed to open grasper jaws.

$E_{\text {close }}$
: Energy needed to close grasper jaws.

$F_b$
: Balancing force.

$F_{i, c}$
: Internal compliant (elastic) tip force.

$F_{i, f}$
: Internal friction force.

$F_{i, h}$
: Iinternal handle force.

$F_r$
: Residual force/absolute balancing error.

$F_{\text {st }}$
: Sensitivity threshold force.

$R_b$
: Relative balancing error.

$x$
: Push-pull rod displacement.

$W_{\text {open }}$
: Work needed to open grasper jaws.

$W_{\text {close }}$
: Work needed to close grasper jaws.

$\eta$
: Mechanical efficiency.

*Clinical and Translational Impact Statement*—Compliant instruments with enhanced haptic sensation have the potential to prevent undetected high pinch forces that can cause tissue damage during laparoscopic surgery.

## I. Introduction

In the last decades, technology that foster laparoscopic or minimally invasive surgery (MIS) developed significantly resulting in a tremendous increase in both the absolute number of MIS procedures as well as different procedure types. For patients, MIS has several advantages compared to open surgery, such as shorter post-operative hospital stay and recovery, less pain and less visible scars [1], [2], [3], [4]. However, surgeons performing MIS experience more discomfort, pain and injuries compared to open surgery, as MIS can be physically and mentally more demanding [5]. A major difference between open and laparoscopic surgery is that with laparoscopic surgery, the intra-abdominal tissue is manipulated using an intermediate instrument, in contrast to open surgery where palpation of tissue by the surgeon’s hands is also possible. When the forces that act on the tissue are distorted due to the internal component interactions and transmissions, the quality of force feedback and haptic feedback that a surgeon can obtain from his hands decreases, potentially influencing performance [6]. In combination with high pressures at the end effector [7], this increases the risk of unintended tissue damage [5].

Several factors contribute to the loss of quality of force feedback. First, most laparoscopic instruments are not designed for optimal haptic feedback to the surgeon’s hand [8]. Second, instrument mechanisms and handles have poor force transmission. In the study of Sjoerdsma et al. [9], the mechanical efficiency was measured of several commonly used laparoscopic instruments. This efficiency was defined as the ratio between the output energy when the jaws are opened as a result of a certain preloading and the input energy supplied to the instrument when the jaws are closing:

\begin{equation*} \eta =\frac {E_{open}}{E_{close}}=\frac {W_{open}}{W_{close}} \tag{1}\end{equation*}

**FIGURE 1.**
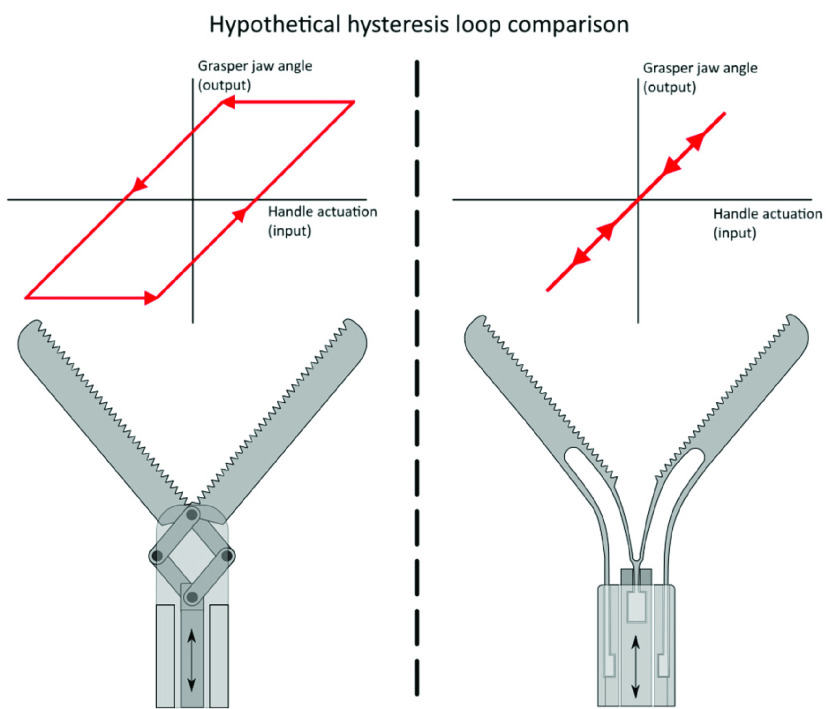
Hypothetical hysteresis loop and mechanism of a conventional grasper tip with low mechanical efficiency (left), and a compliant grasper tip with extremely low hysteresis and a mechanical efficiency of ~100% (right).

This study showed that mechanical efficiency ranged from 8% to 42%, indicating that during an opening and closing cycle, more than half of the input energy is lost. This can be explained by the working principle of conventional laparoscopic instruments. Conventional laparoscopic instruments utilize a 4-bar linkage mechanism combined in a mechanism to transfer control forces from the handle to the end effector [10]. The connecting parts of this mechanism can be seen as sliding bearings, which need some play to be able to rotate relative to each other without too much friction. Moreover, in these instruments a ball and socket joint is used to transfer handle forces, which also adds to the mechanical play. This play appears to be very limited, because of the small size of the mechanism. However, the large arm of an instrument handle magnifies the play perceived by the surgeon, resulting in significant hysteresis between the handle actuation by the surgeon (input) and grasper jaw angle (output) (Fig. 1). Therefore, the surgeon also has to rely on visual feedback to determine how the applied input force on the handle is being applied to the targeted tissue, or whether the instrument is still in the range of the play. This problem becomes more evident during sensing of delicate tissue handling when high internal friction and thus low mechanical efficiency $\eta $ in the instrument can prevent small force perception completely [11]. In that case, the grasper’s sensitivity threshold force $F_{st}$-defined as the smallest grasper force perceivable through the instrument handle - of conventional laparoscopic graspers is too high. Lowering this threshold (while maintaining force perception for larger forces) can potentially prevent undesired tissue damage, but also open possibilities towards a more precise tissue assessment through delicate grasping, which can be helpful in e.g. metastasis recognition.

In earlier studies [12], [13], [14], a monolithic and compliant laparoscopic grasper tip has been described that showed a hysteresis close to zero when opening and closing of the tip under different loading conditions, potentially offering a solution for delicate grasping and small for perception. The compliant tip (Fig. 1) uses flexible elements to open and close the grasper, rather than conventional tips which use pin and joint connections. Similar to a conventional grasper tip, a push pull rod with displacement $x$ is used to transfer the handle movement to the compliant grasper tip. Monolithic compliant mechanisms are also easier to clean and sterilize than conventional bar link mechanisms, as all surfaces of the mechanism prone to contamination are exposed. As the compliant tip does not have any mechanical play it has the potential to restore some of the haptic feedback during laparoscopic surgery. However, in the aforementioned papers [5], [6], the compliant tip was not integrated into a clinically feasible instrument, because balancing the relatively high forces required to open the tip proved to be challenging. These internal compliant forces ( $F_{i,c}$) are introduced by the elastic deflection of the compliant elements of the grasper tip. If these internal instrument forces are not balanced out by a balancer, during laparoscopic surgery they would be perceived by the surgeons through the grasper handle. As $F_{i,c}$ is almost indistinguishable from tissue forces $F_{t}$ which are perceived through the same handle interface, an unbalanced compliant grasper would distort the tissue force perception. Therefore, a significant majority of the internal compliant forces should be balanced out. In this paper, a design and first prototype for a magnetically balanced compliant laparoscopic grasper is presented. From the produced prototype, the hysteresis and mechanical efficiency was measured, and the haptic feedback restoration was evaluated by comparing the force perturbation sensitivity of the compliant grasper to conventional graspers.

## II. Materials and Methods

### A. Grasper Design

The Delft Design Method [15] was used to structure the design process with a special focus on Component interaction and function interaction as seen in the Bare Minimum Design- Component Interation Analysis (BMD-CIA) method [16]. The study goal is to come up with a design that improves safe tissue handling while grasping. For safe execution of precision tasks such as grasping delicate tissue, high mechanical efficiency and low internal friction are crucial [11]. Therefore, these were key principles throughout the design process. As shown before, compliant mechanisms provide promising results to achieve this [12], [13], [14]. Therefore, not only the grasper tip but also the handle design will utilize the compliant mechanism concept.

A list of design requirements was established, taking into account clinical aspects. These requirements were determined by a user committee, consisting of laparoscopic surgeons and mechanical engineers:

1. All internal elastic forces $F_{i}$ should be balanced for at least 80% (i.e. a maximum balancing error of 20%), and at most 100% to prevent overbalancing;
2. Mechanical efficiency $\eta $ should be over 50%;
3. During grasping, the compliant grasper should enable the user to also perceive subtler, smaller grasping forces than conventional graspers. Therefore, the sensitivity threshold force $F_{st}$ of the compliant grasper should be at least 30% lower compared to conventional graspers;
4. The grasper should be intuitive to use as indicated by user feedback;
5. The instrument should be able to undergo 150 working cycles (based on number of grasping actions in laparoscopic colectomies, a procedure with a high number of grasping actions [17]) before it needs rebalancing;
6. The instrument should be suitable for atraumatic grasping during laparoscopic surgery with 5mm diameter instruments;
7. The maximum weight of the instrument is 300 grams.

The functional tip design of the novel compliant laparoscopic grasper is similar to a conventional laparoscopic grasper. The compliant tip was fabricated with wire-EDM from superelastic Nitinol.

In order to meet the balancing requirement, a balancing mechanism should be integrated in the instrument design. To determine the necessary balancing force characteristics ( $F_{b}$), the force-displacement curve of the standalone compliant tip must be characterized. This was done by measuring the opening and closing cycle of the compliant tip on a custom built linear stage coupled with a force sensor (KD24s 50N, ME-Meßsysteme GmbH, Germany) mounted on the push pull rod of the compliant tip. The cycle was repeated 70 times.

**FIGURE 2.**
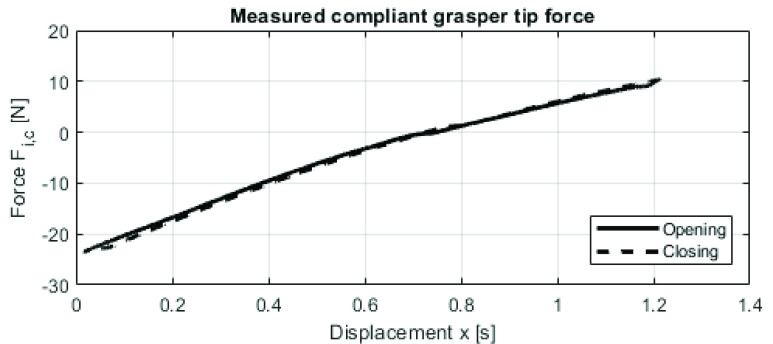
Force-displacement measurement of the compliant tip (standalone). The grasper tip is fully opened at x=−0.75 mm, and x=0.45 mm is the fully closed position.

The resulting force-displacement curve of the standalone tip is shown in Fig. 2. The maximum compliant tip force $F_{i,c}$ is $-23.5N$. It can be seen that the curve is almost linear, which means that the balancing mechanism needs a linear force-displacement curve. From these measurements, the mechanical efficiency of the standalone compliant tip was calculated at 96%. Based on the force measurement results, Requirement 1 was quantified and a novel balancer mechanism was designed.

In previous designs, a compliant balancing mechanism with preloaded springs was used [12], [13], [14]. However, tuning this mechanism proved to be a challenge due to a high sensitivity to temperature. In addition, the high forces required for pre-loading of the balancer required complex shapes of the stiff spring elements to prevent excessive material strain and plastic deformation

**FIGURE 3.**
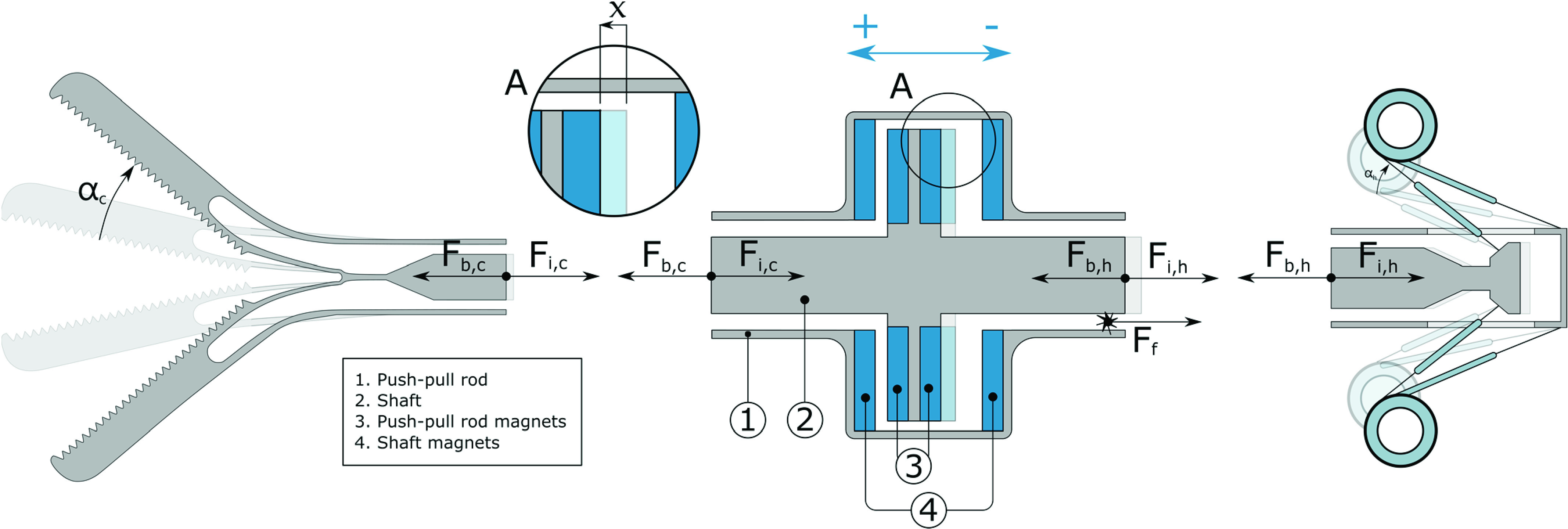
Schematic cross sections of the balanced compliant grasper concept. From left to right: compliant grasper tip, magnetic balancing mechanism and handle. Contact friction forces $F_{f}$ (e.g. between the (1) push-pull rod (1) and shaft (2)) are visualized, as well as internal forces $F_{i}$ and balancing forces $F_{b}$, both divided in a tip part (suffix $c$) and a handle part (suffix $h$). The positive push-pull rod displacement ( $x$) is indicated with an arrow in cut-out A. The blue elements depict the ring-shaped balancing magnet pairs: one magnet pair mounted to the push pull rod (3), and one magnet pair connected to the outer shaft and housing (4). The blue arrow indicates the magnets’ magnetization direction.

To overcome these issues, a magnetic balancing mechanism was designed. It consists of two sets of neodymium magnet rings with the opposite magnetic poles facing each other (Fig. 3). One set of magnets is connected to the outer housing, while the other set is mounted to the instrument’s push pull rod. This results in a linear balancing force $F_{b}$ displacement curve. Using the magnetic field modelling program Finite Element Method Magnetics (FEMM) the magnetic rings were dimensioned such that $F_{b}$ is equal and opposite to the sum of internal forces:

\begin{equation*} F_{b}\left ({x }\right)=-\sum {F_{i}(x)} \tag{2}\end{equation*}

The FEMM model was verified in a force test setup with similar magnet arrangement and proved to be 98% accurate. This model was used to choose a suitable magnet configuration and magnet size to optimize for the required balancing force. The magnetic balancer is integrated into the handle design and designed to be adjustable in order to modify the balancer force during prototype testing.

The handle design features a tweezers shape stimulating a pinch grip using only the thumb and index finger tips. In this way, the most sensitive parts of the user’s hand [18] will be used to control the instrument and receive haptic feedback. The tweezers’ joints are compliant, thus minimizing handle hysteresis. However, this introduces an internal compliant handle force $F_{i,h}$, additional to the compliant tip force $F_{i,c}$ that need to be balanced as well. $F_{i,h}$ was estimated using a non-linear Solidworks Simulation deformation model. This means that the ideal required balancing force is:

\begin{equation*} F_{b}\left ({x }\right)=-\sum {F_{i}(x)} =-\left ({F_{i,c}\left ({x }\right)+F_{i,h}(x)+F_{i,f} }\right) \tag{3}\end{equation*}

With $F_{i,f}$ being the internal friction force that, unlike a conservative, elastic force cannot be balanced out completely by a balancing mechanism. As the evaluations of Requirement 1 and 2 showed that all internal forces are relevant, $F_{i,f}$ was included in Equation 3 as well.

### B. Balancing and Mechanical Efficiency

Technical validation of the instrument was done to evaluate the hysteresis of the instrument. This also validates Requirement 1: the balancing of at least 80% and at most 100% of the internal forces $F_{i}\left ({x }\right)$ and Requirement 2: a minimum mechanical efficiency of 50%. According to Requirement 1, the balancing requirement to be fulfilled is:

\begin{equation*} 0.8\sum {F_{i}(x)} \le F_{b}(x) < \sum {F_{i}(x)} \tag{4}\end{equation*}

The internal forces are a summation of $F_{i,c}$, $F_{i,h}$ and $F_{i,f}$, according to Equation 3. $F_{i,c}$ was measured as a function of $x$ and $F_{i,h}$ was estimated. Although the balancing force $F_{b}$ cannot be measured directly either, it can be calculated by first measuring the residual force $F_{r}$ (also known as the absolute balancing error). This is the internal force that is still present when balancing is applied:

\begin{equation*} F_{r}\left ({x }\right)=F_{i,c}\left ({x }\right)+F_{i,h}\left ({x }\right)+F_{b}(x)+F_{i,f} \tag{5}\end{equation*}

$F_{i,f}$ can be estimated by calculating the difference in residual force between the opening and closing cycle of the hysteresis loop:

\begin{equation*} F_{i,f}=F_{r,open}-F_{r,close} \tag{6}\end{equation*}

Using Equation 5, 6 and 7, and the measurement of $F_{r}$, $F_{b}$ can be calculated:

\begin{equation*} F_{b}\left ({x }\right)=F_{r}(x)-\left ({F_{i,c}\left ({x }\right)+F_{i,h}\left ({x }\right)+F_{i,f}(x) }\right) \tag{7}\end{equation*}

**FIGURE 4.**
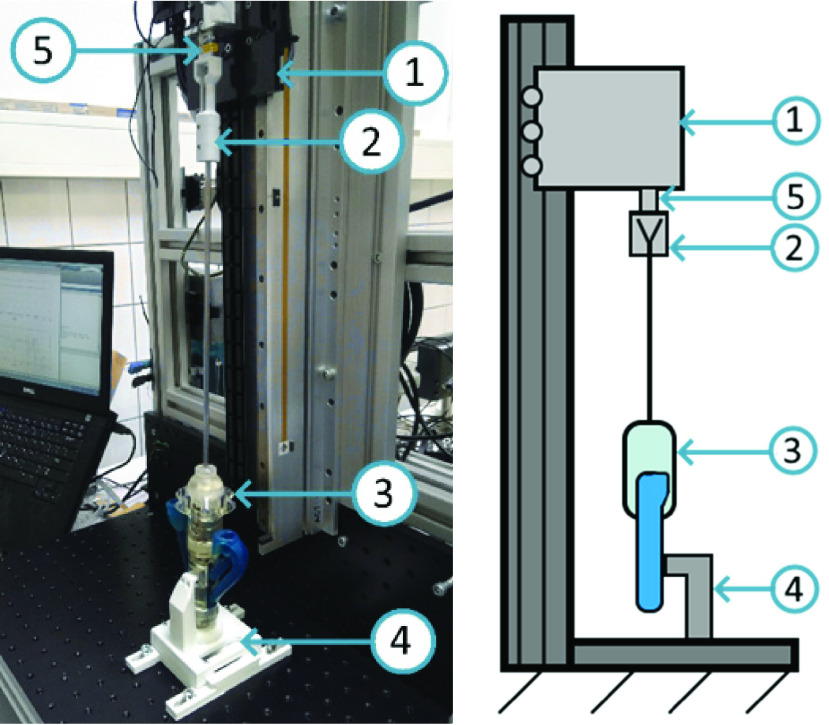
Balancing error experimental setup, with linear stage (1), sensor mount (2), instrument (3), instrument mount (5) and force sensor (5).

In the results, Equation 7 will be used in Equation 8 to calculate the relative balancing error to evaluate Requirement 1. With $F_{r}$ measured for the complete opening and closing cycle of the grasper jaws, also $\eta $ can be calculated using Equation 1 to validate Requirement $2.~F_{r}$ was measured six times at the instrument’s push-pull rod, without any load on the grasper tip. The instrument was clamped at the shaft near the grasper tip to a force sensor (LSB200, Futek, USA), which in turn was mounted to a linear stage (ACT115 Aerotech, USA). The push pull rod was fastened to an adjustable platform, near the compliant handle (Fig. 4).

To be able to compare $\eta $ of the novel compliant grasper and conventional laparoscopic graspers, the force needed to open and close the grasper jaws of a unbranded grasper with considerable friction and mechanical play and a branded commercial grasper (Aesculap PL055R, Aesculap AG, Germany), was measured as well. This was done on the same experimental setup as the novel compliant grasper.

The data was processed with a custom made script (Supplemental A) using MATLAB (R2019b, The Mathworks, Inc., Natick, Massachusetts, USA) to distinguish between closing and opening force measurements of the compliant tip.

### C. Quality of Haptic Feedback

Quality of haptic feedback was defined in terms of the ability of a laparoscopic grasper to enable the surgeon to perceive a load force on the grasper tip that is as small as possible. This is a favorable characteristic during laparoscopic procedures where small forces and force differences are relevant. The combined effect of hysteresis and friction as well as the ergonomic handle design largely determines the quality of haptic feedback. In order to validate the quality of haptic feedback, the sensitivity threshold force $F_{st}$ of two conventional laparoscopic graspers and the compliant grasper was compared.

**FIGURE 5.**
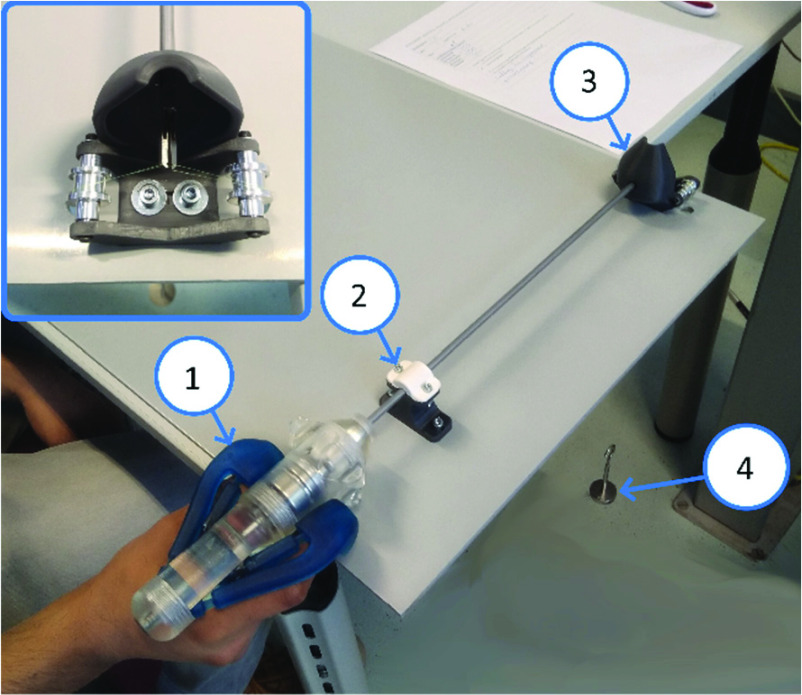
Sensitivity threshold force experimental setup, with instrument (1), instrument clamp (2), visual shielding and load application (3) and weight (4).

A test setup was designed and built (Fig. 5). A load was applied to the grasper tip using pulleys, nylon strings and calibrated weights of 50 grams each. The instruments were clamped and the instrument tip was shielded from the participants so that it was not visible, to ensure that the participants only relied on haptic feedback and not on visual feedback. Three instruments were used in this study: the balanced grasper, and two conventional laparoscopic graspers; an unbranded grasper with considerable friction and mechanical play, and a branded commercial grasper (Aesculap PL055R, Aesculap AG, Germany).

Ethical approval (approval code 2587) was obtained at the Delft University of Technology (TU Delft) Human Research Ethics Committee (HREC). Twenty five participants were selected according to the following criteria: 18–65 years old and having no surgical experience. Prior to the experiment, the participants gave informed consent. They were asked to hold the instrument handle, using their dominant hand, squeezing the handle hard enough to keep the handle in the same (almost closed) position. Using weights and pulleys, an increasing gravitational force was applied to the grasper tip via nylon strings. At the start of the experiment there was already a load of 50 grams to preload the string. The increment of weight was 50 grams as well. As soon as the participant felt the haptic feedback of this load force in the handle, he or she was instructed to notify the experimenter. This load was named and stored as the sensitivity threshold force $F_{st}$. The experiment ended at this point, and was repeated with the remaining instruments in randomized order.

### D. Questionnaire

After the sensitivity threshold experiment, a questionnaire was conducted with the participants. First, they were asked which instrument handle was more comfortable to use: the tweezers handle, the traditional grasper handle or equally comfortable. In the second (open) question, the participants were asked to provide suggestions on grasper design improvement from a user’s perspective regarding ergonomics, weight, center of gravity and balancing. Participants were allowed to give multiple answers to this question.

### E. Expert Opinion

An expert gastrointestinal laparoscopic surgeon with 19 years of experience used the compliant grasper in a ForceSense laparoscopic box trainer (MediShield, Delft, The Netherlands). He performed basic handling tasks such as peg transfer. After this, an expert opinion on the balanced instrument was asked.

## III. Results

### A. Balanced Grasper Prototype

**FIGURE 6.**
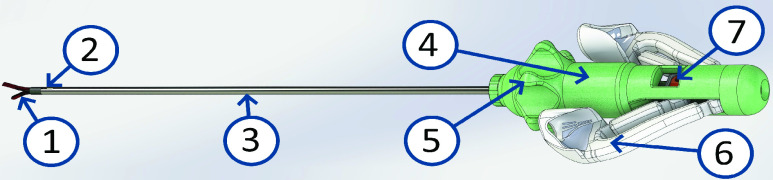
Compliant instrument design overview, with grasper tip (1), tip locking mechanism (2), shaft (3), balancing mechanism housing (4), rotating interface (5), handle (6) and push pull rod locking (7). Note that the used colors do not represent the final prototype.

**FIGURE 7.**
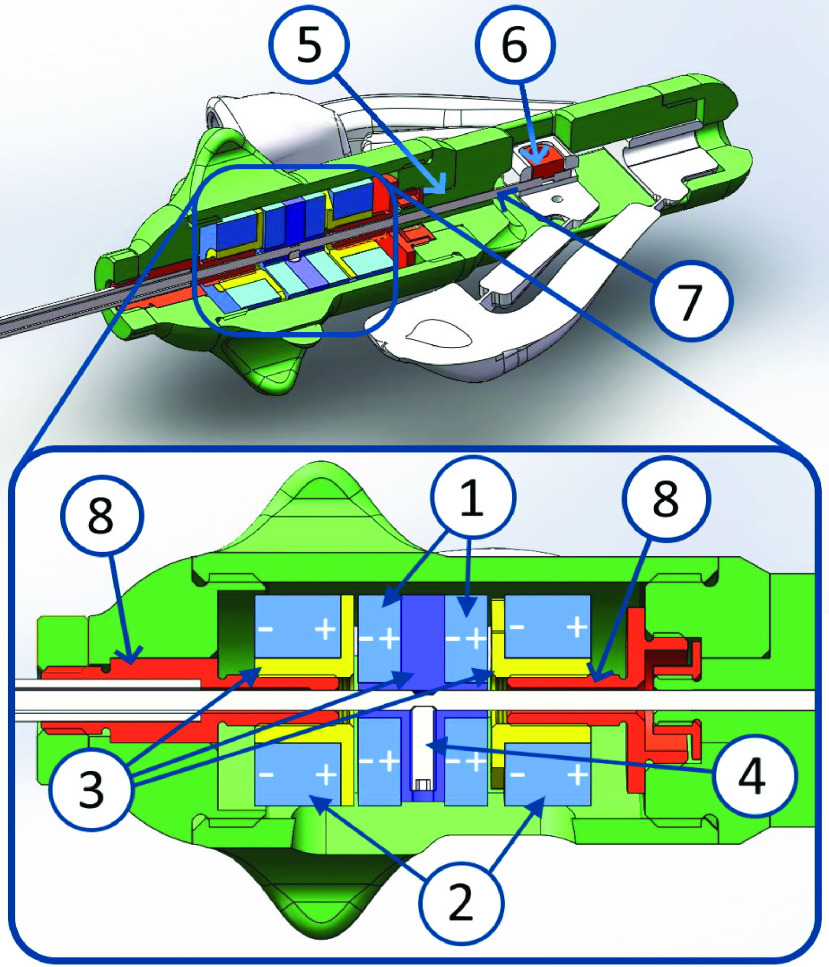
Balancing mechanism and handle cross Section view, with push pull magnet pair (1), housing magnet pair (2), magnet mounts (3), screw pin (4), grasper rotation joint (5), push pull rod locking (6) and push pull rod (6). The magnetization direction of the magnets are noted with the (+) and (−).

**FIGURE 8.**
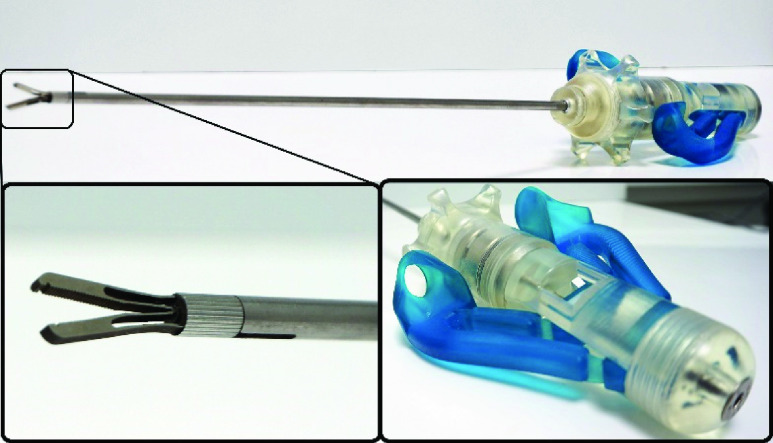
Balanced compliant laparoscopic grasper prototype, with grasper tip on the left (bottom), balancing mechanism housing, rotating interface, tweezers handle on the right (bottom).

The designed balanced grasper is shown in Fig. 6, 7 and 8. The balancing mechanism is integrated in the knob used for longitudinal rotation of the instrument tip. While the grasper tip was made from superelastic Nitinol grasper tip, the shaft, push-pull rod and other metal parts were made from stainless steel (AISI316). The balancer housing and tweezers handle parts were 3D-printed from a photopolymer resin (Standard clear resin, Formlabs Inc., Somerville, MA). The handle flexures were cut from 0.1mm thick spring steel and bonded to their respective tweezers handle parts to ensure a hysteresis free handle actuation.

**FIGURE 9.**
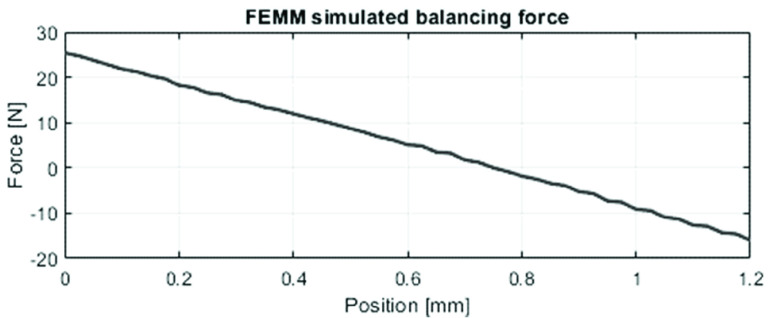
Simulated balancing force $F_{b}(x)$ at its working point.

The neodymium N45 balancer magnets were modelled in FEMM to have a maximum balancing force $F_{b}$ of $25.4N$ at a $x=0.0mm$, decreasing linearly to $F_{b}=0$ at $x=0.75mm$ (Fig. 9). To prevent undesired radial magnetic forces, the push pull rod was centred by integrating sliding contact bearings.

The balanced grasper prototype was able to grasp and rotate about its longitudinal axis simultaneously. With balancing enabled, grasping took significantly less perceived effort compared to grasping without a balancing force. The instrument was able to undergo 120 opening and closing cycles without the need for rebalancing. The instrument is suitable for 5mm laparoscopic surgery and grasper weighs 273 grams.

### B. Balancing and Mechanical Efficiency

**FIGURE 10.**
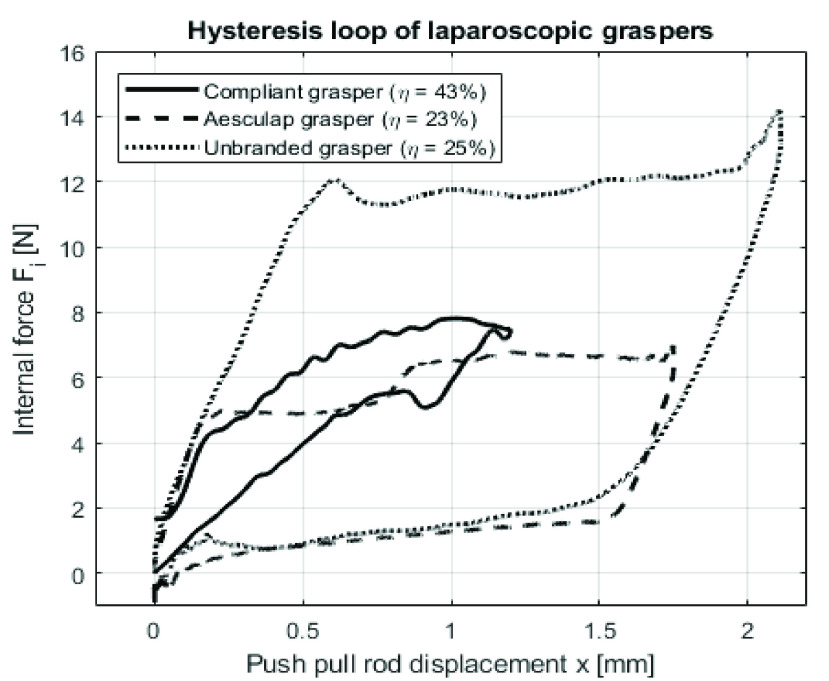
Hysteresis loops of the balanced grasper, Aesculap and unbranded grasper, expressed is internal force ( $F_{i}$). In the legend, the respective mechanical efficiencies are indicated.Mean residual force $F_{i}$ as a function of push-pull rod displacement $x$.

The maximum measured residual force $F_{r}$ of the novel grasper was $7.9N$ at push-pull rod displacement of $x=1.0mm$ (during the opening cycle), which is the fully opened position of the grasper tip. The residual force over the complete working range (from fully opened to fully closed can be found in Fig. 10.

Using the hysteresis loop data from the experiment, the compliant tip force $F_{i,c}$ measurements (Fig. 2) and compliant handle force $F_{i,h}$ FEA simulations, and Equation 7 to calculate the balancing force $F_{b}$, the relative balancing error $R_{b}$ from design Requirement 1 can be calculated:

\begin{equation*} R_{b}=\frac {F_{b}\left ({x }\right)}{F{}_{i,c}\left ({x }\right)+F_{i,h}\left ({x }\right)+F_{i,f}} \tag{8}\end{equation*}

**FIGURE 11.**
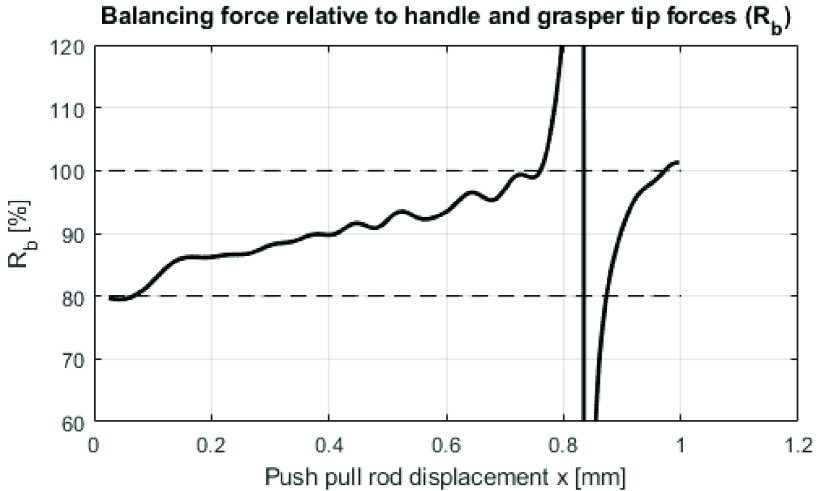
Relative balancing error $R_{b}$ of the combined (mean) instrument opening and closing cycle. The dashed lines represent the minimum and maximum balancing error, as stated in Requirement 1.

The calculation results over the whole working range $x$ of the instrument are shown in Fig. 11. Over the majority of the working range, the internal forces are balanced between 80% and 100%. Around the neutral point $\left ({x=0.75mm }\right)$, $R_{b}$ is theoretically infinity because $F{}_{i,c}(x)+F_{i,h}\left ({x }\right)+F_{i,f}$ are (close to) zero, therefore around the neutral point Equation 8 becomes invalid, but the balancer remains stable.

In Figure 10, the residual force measurement shows a hysteresis loop characteristic of the novel grasper. Therefore, using Equation 1, the mechanical efficiency can be calculated from the measurement data, using Equation 6 to estimate the friction force $F_{i,f}$. The maximum estimated friction force was $2.7N$ at $x=-0.75mm$ for the balanced compliant grasper. The mechanical efficiency of the balanced compliant grasper $\eta $ is 43%.

The maximum measured friction force $F_{i,f}$ was $6.8N$ and $14.1N$for the Aesculap and unbranded conventional laparoscopic grasper, respectively.The hysteresis loops of both conventional graspers can be found in Fig. 10. Using this data, the mechanical efficiency $\eta $ of the unbranded and Aesculap graspers was calculated at 23% and 25%, respectively. This means that the balanced compliant grasper has a higher mechanical efficiency than both conventional graspers.

### C. Quality of Haptic Feedback

**FIGURE 12.**
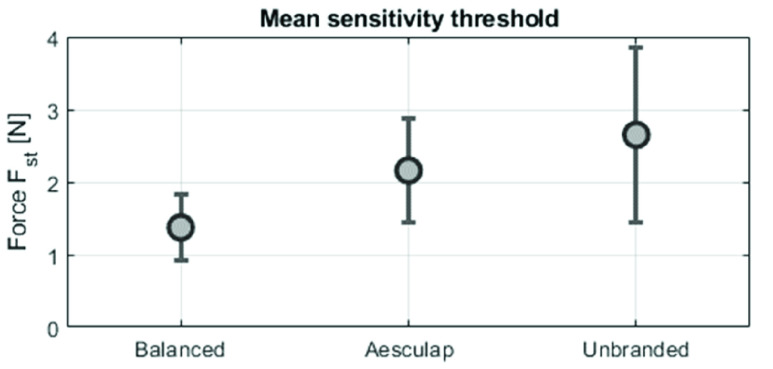
Sensitivity threshold force measurement (mean and SD) of three different laparoscopic instruments (n=25).

The sensitivity threshold force $F_{st}$ was measured with 25 participants. It was the lowest for the novel balanced compliant grasper (mean $1.37N$, SD $0.44N$), followed by the Aesculap grasper (mean $2.\mathrm {d}15N$, SD $0.71N$) and the unbranded grasper (mean $2.65N$, SD $1.20N$). See also Fig. 12. Comparing the sensitivity threshold force of the balanced grasper to the conventional graspers separately using an ANOVA test yielded the p-values < 0.001. This shows that the balanced compliant grasper has a significantly lower threshold force compared to both low and high quality conventional laparoscopic graspers. Furthermore, the mean sensitivity threshold force of balanced compliant grasper the is 36% lower than the best performing conventional grasper (Aesculap). Ten out of 25 participants perceived a load difference after applying the first load, whereas only two participants perceived the same with the conventional graspers. All measurement results can be found in supplemental B.

### D. Questionnaire

In the questionnaire, 24% of the participants found the tweezers handle of the novel grasper the most comfortable to use, 68% preferred the traditional handle, whereas 8% had no preference. As for design improvements, participants had the most remarks on the counter-intuitive handle ergonomics (11), followed by the centre of gravity being too far from the hand holding the instrument (7). Instrument weight was deemed too high by six participants, whereas two participants noted that the low friction was perceived as unfamiliar. All questionnaire results can be found in supplemental B.

### E. Expert Opinion

The surgeon acknowledged the potential of having an hysteresis free instrument in order to improve haptic feedback. However, he mentioned that the ergonomics should be improved in order to assess the full feedback restoring potential of the instrument. The surgeon suggested that the handle grip add on might solve this problem by separating the working lines of the control force and the holding force. Also, he noted that the balanced compliant grasper would be more comfortable to hold if its center of gravity is closer to the user’s hand.

## IV. Discussion

The balanced compliant grasper prototype proved that magnetic balancing is a viable technique to reduce the perceived internal actuation forces of compliant mechanisms with more than 80 but less than 100% percent. This means that Requirement 1 is met. The overall mechanical efficiency $\eta $ of the balanced compliant grasper is 43%, meaning that Requirement 2 ( $\eta >50\%$) is not met. Also, the prototype lowers the mean sensitivity threshold force with 36%, which means that Requirement 3 is met. The lower sensitivity threshold potentially enables surgeons to perceive smaller, more subtle forces compared to conventional laparoscopic graspers. The results of the questionnaire and expert opinion show that the instrument’s ergonomics can be improved in terms of center of gravity, weight and intuitive use. Therefore, Requirement 4 is not fully met. Requirement 5 (cycle number without need for balancing), Requirement 6 (5mm laparoscopy suitability) and Requirement 7 (maximum weight) were met as well.

### A. Instrument Design

Over the majority of the working range, the internal forces are balanced between 80% and 100%. The overall mechanical efficiency was lower than expected. Taking into account the high mechanical efficiencies of the standalone compliant grasper tip (95%) and magnetic balancing mechanism (known to have a mechanical efficiency close to 100%), it can be concluded that the internal friction force at the sliding bearing surfaces is the main cause of the low mechanical efficiency. It was expected that the efficiency of the novel instrument would have been higher. The mechanical efficiency of the unbranded and Aesculap graspers was calculated at 23% and 25%, respectively. This means that with a mechanical efficiency of 43%, the novel grasper is still outperforming existing graspers. The cause of the low mechanical efficiency is the friction force present in the novel instrument. As presented in Equation 6, the estimated friction force is relatively small compared to the conventional graspers. However, reducing the remaining friction could give the novel grasper an even larger advantage, which should be a focus point of a next prototype design process.

The added cost of a magnetic balancing mechanism is relatively low, as it consists of simple parts that can be easily machined on a 3-axis lace, while the magnets are commercially available. The balancing mechanism is modifiable over a wide range (75% reduction relative to maximum balancing force) by simply rotating the proximal magnets. Although the treaded connection inside the magnet facilitates balancer force adjustment during prototype testing, this adjustment option requires and increases manufacturing cost and reprocessing complexity and might not be needed in a clinical context. During further development aimed at a mass produced balanced grasper, it should be investigated whether a fixed, non-adjustable mechanism does not significantly reduce balancing performance during the instrument’s life cycle.

When considering a broader engineering perspective, application of compliant grasping mechanisms should be considered in robotic surgery. Compared to the current state of the art cable and pulley systems, compliant mechanisms are easier to clean and sterilize and do suffer less from wear and tear. Implementation of compliant mechanisms in robotic surgery might increase the number of reprocessing cycles that robotic instruments can undergo, reducing surgery costs and environmental footprint. In general, compliant mechanisms and magnetic balancing combined can be considered in engineering challenges in (tele-)manipulation where hysteresis and friction should be minimized.

### B. Quality of Haptic Feedback

The ANOVA analysis showed that the balanced compliant grasper has a significantly higher sensitivity to force perturbations during laparoscopic surgery than conventional laparoscopic graspers. The mean sensitivity threshold force $F_{st}$ of the balanced compliant grasper is 36% lower than the best performing conventional grasper (Aesculap).

It should be noted that with the novel balanced compliant grasper, 10 out of 25 participants already perceived a load difference after applying the first load, whereas only two participants perceived the same with the conventional graspers. This means that the sensitivity threshold difference between the novel and conventional graspers is possibly even larger at lower force levels. Also, the increment was only done with weights of 50 grams. Measuring with smaller weight increments can provide more detailed information on the quality of haptic feedback.

In laparoscopic surgery, tissue grasping tasks can take many forms. Roughly, two task types can be discriminated: the first category is related to the execution of the primary intended surgical task, like removing malignant tissue from healthy tissue or metastasis palpation where it is important for the surgeon to be able to receive reliable haptic feedback about tissue stiffness and texture. Secondly, there is the secondary task of surgical preparation, which includes the less subtle grasping, manipulation and dividing of tissue in order to reach and prepare a certain site in the patient’s body. When both task types are performed simultaneously (e.g. passive tissue grasping to create an operating space while removing a tumour), the secondary task is executed less consciously and surgical performance drops [19]. Here, improved haptic feedback would be beneficial for secondary task performance as well, in terms of excessive grasping force or tissue grasping slip. Therefore, for both task types, it should be investigated whether the novel feedback-restoring compliant grasper can improve haptic feedback. It should also be investigated whether the novel instrument can enable surgeons to perceive pressure differences such as blood vessel pulses through delicate grasping.

This study focused on the proof of concept of a statically balanced compliant laparoscopic grasper and it technical and clinical feasibility. The sensitivity threshold force measurements show the potential for compliant mechanisms to improve quality of haptic feedback. However, there are much more perspectives regarding haptic feedback. Therefore, in-depth validation of haptic feedback in relation to other (tractive) interaction forces, is needed of the instrument inside a validated box trainer task in order to measure performance in a clinically relevant setting [6], [20]. Also, other boundary conditions for clinical conditions should be met, such as the ability to disassemble and assemble the instrument for cleaning and inspection [16] and how the components withstand the cleaning and sterilisation processes. Precise tuning the magnets to ensure optimal balancing force also was a challenge that should be addressed in a next version of the instrument.

### C. Questionnaire

The results of the questionnaire showed that the weight, centre of gravity and handle ergonomics need to be optimized. Although Requirement 7 (maximum weight) has been met, in the questionnaire several remarks were made about the weight of the instrument. Moreover, majority of the participants preferred the traditional handle of the conventional graspers over the tweezers handle of the balanced compliant grasper. Also, during experiment instruction, it became clear that participants often needed a short demonstration to use the tweezers handle, whereas this was not the case with the conventional pistol handle.

### D. Expert Opinion

The surgeon’s comments on the handle ergonomics showed again that the prototype design should be improved to accommodate for intuitive use, optimized for delicate grasping. As suggested by the surgeon, a pistol grip was added in a follow-up pilot design study, the instrument handle was more comfortable and intuitive to use, according to a surgeon. This will be taken into account when designing the next balanced compliant grasper prototype.

## V. Conclusion

This study shows that the novel balanced compliant grasper can improve the quality of haptic feedback compared to conventional laparoscopic graspers. The instruments’ mechanical efficiency is much higher compared to conventional laparoscopic graspers and smaller forces can be perceived, which could improve delicate tissue grasping. The questionnaire and expert opinion showed that redesign of the novel instrument is needed to improve the ergonomics is needed. Also, the design should be optimized for cleaning of all instrument components.

## Supplementary Materials

Supplementary materials
