# Supplementary material for: Design and Evaluation of a Balanced Compliant Laparoscopic Grasper
Source: IEEE J Transl Eng Health Med. 2023 Jul 3;11:451–9. doi: 10.1109/JTEHM.2023.3291925 (PMC10561751; doi:10.1109/JTEHM.2023.3291925)
Supplement: Supplementary materials [file jtehm-3291925-mm.zip › README.pdf]

DESCRIPTION: runme.m is the main file that runs all Matlab files associated with Supplemental A of the paper "DESIGN AND EVALUATION OF A BALANCED COMPLIANT LAPAROSCOPIC GRASPER". Run this file to load and process the raw data and reproduce all figures in the paper. Matlab is needed to run the scripts. Supplemental B is a spreadsheet (Excel) file that contains the raw data of the sensitivity and questionnaire results.

SIZE: The total size of the file 1.36 MB

PLAYER INFORMATION: coding file

PACKING LIST: jtehm-3291925-mm.zip

CONTACT INFORMATION:

Jan-Willem Klok
PhD Candidate
Technische Universiteit Delft
Department of BioMechanical Engineering
3mE Room F-0-200
06 20899101
Email: j.w.a.klok@tudelft.nl
