# Supplementary figures and images for: Design and Evaluation of a Balanced Compliant Laparoscopic Grasper
Source: IEEE J Transl Eng Health Med. 2023 Jul 3;11:451–9. doi: 10.1109/JTEHM.2023.3291925 (PMC10561751; doi:10.1109/JTEHM.2023.3291925)

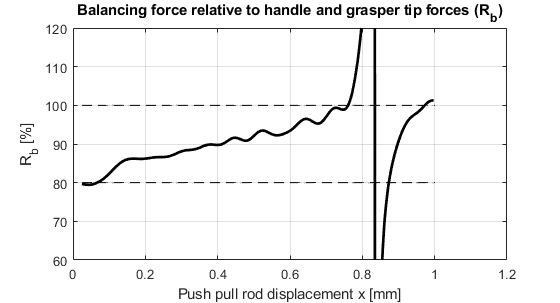

Supplement: Supplementary materials [file jtehm-3291925-mm.zip › Supplemental A - Matlab scripts/figure 11/figure11.png]

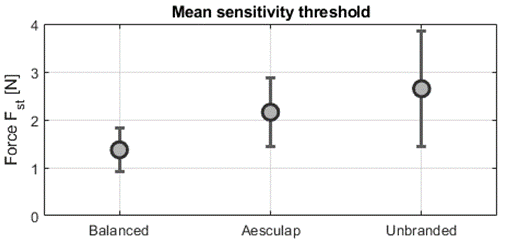

Supplement: Supplementary materials [file jtehm-3291925-mm.zip › Supplemental A - Matlab scripts/figure 12/figure12.png]

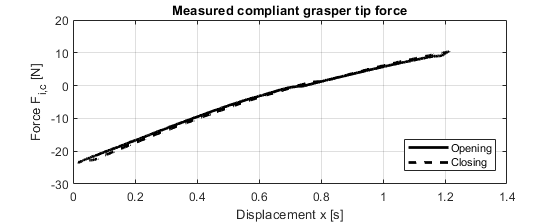

Supplement: Supplementary materials [file jtehm-3291925-mm.zip › Supplemental A - Matlab scripts/figure 2/figure2.png]

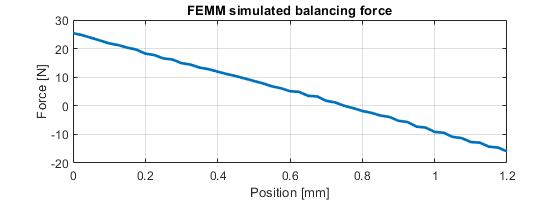

Supplement: Supplementary materials [file jtehm-3291925-mm.zip › Supplemental A - Matlab scripts/figure 9/figure9.png]
